# HYA ameliorated postprandial hyperglycemia in type 1 diabetes model rats with bolus insulin treatment

**DOI:** 10.1007/s00592-025-02459-6

**Published:** 2025-02-03

**Authors:** Yuta Yamamoto, Katsuya Narumi, Naoko Yamagishi, Yasunori Yonejima, Ken Iseki, Masaki Kobayashi, Yoshimitsu Kanai

**Affiliations:** 1 Department of Anatomy and Cell Biology, Graduate School of Medical and Pharmaceutical Sciences, Wakayama Medical University, 811-1 Kimiidera, Wakayama, 641-8509 Japan; 2 Laboratory of Clinical Pharmaceutics and Therapeutics, Division of Pharmasciences, Faculty of Pharmaceutical Sciences, Hokkaido University, Sapporo, Japan; 3 Noster Inc., Kyoto, Japan

**Keywords:** HYA, Postprandial hyperglycemia, GLP-1, CCK, Type 1 diabetes mellitus

## Abstract

**Aims:**

The oral administration of linoleic acid immediately before glucose tolerance test (OGTT) ameliorated postprandial hyperglycemia via GPR120 pathway in normal and type 1 diabetes (T1DM) rats. Linoleic acid could promote inflammatory mediators, but 10-hydroxy-*cis*-12-octadecenoic acid (HYA) converted from linoleic acid by *Lactobacillus plantarum* has higher GPR120 agonistic activity without promoting inflammatory mediators. This study examined whether the oral-administration of HYA immediately before OGTT also ameliorated the postprandial hyperglycemia in normal rats and T1DM rats injected with bolus insulin.

**Methods:**

Normal and T1DM male Sprague-Dawley rats received HYA immediately before OGTT. Other T1DM rats were given HYA and Humulin R immediately before OGTT. We measured the concentration of glucose, insulin, glucagon-like peptide 1 (GLP-1) and cholecystokinin in blood before and after OGTT. We also measured the amount of glucose in the gastric tract after OGTT, and the amount of uptake of methyl-α-D-glucopyranoside in CACO-2 cells.

**Results:**

Postprandial hyperglycemia was ameliorated by HYA in normal rats, and the postprandial blood glucose levels were slowly elevated by HYA in the T1DM model rats. HYA partially inhibited the uptake of methyl-α-D-glucopyranoside in CACO-2 cells. HYA slowed gastric motility and increased the plasma GLP-1 and cholecystokinin levels in normal rats. HYA also ameliorated the postprandial hyperglycemia in T1DM rats given bolus insulin.

**Conclusion:**

Oral administration of HYA immediately before OGTT ameliorated postprandial hyperglycemia through inhibition of glucose absorption and slowing of gastric motility in normal rats. Furthermore, this beneficial effect of HYA was also revealed in T1DM rats injected with bolus insulin.

**Supplementary Information:**

The online version contains supplementary material available at 10.1007/s00592-025-02459-6.

## Introduction

Fatty acids are a source of energy that are absorbed in the small intestine after digestion of pancreatic lipase. Some fatty acids are sensed by G protein-coupled receptors in the intestinal mucosa [1], and long-chain fatty acids are agonists of G protein-coupled receptor (GPR) 40 and/or GPR120 [2]. Oral administration of linoleic acid, included in long-chain fatty acid, two hours before an oral glucose tolerance test (OGTT) reportedly ameliorated postprandial hyperglycemia by promotion of insulin secretion via GPR40 pathway in mice [3]. We previously demonstrated that orally-administered linoleic acid immediately before an OGTT slowed the gastric motility through promoting GLP-1 secretion via GPR120 pathway, which ameliorated the postprandial hyperglycemia in normal rats and slowed the elevation of postprandial glucose level in type 1 diabetes model rats [4]. Linoleic acid is also converted to lipid mediators of the inflammatory responses including prostaglandins and thromboxane through arachidonic acid cascade [5]. The concentration of prostaglandin E2 is increased in the ileum and white adipose tissue of the mice by the administration of linoleic acid. Long-term use of linoleic acid to ameliorate postprandial hyperglycemia might therefore be inappropriate because of the production of lipid mediators.

*Lactobacillus plantarum* was reported to hydroxylate a double bond in polyunsaturated fatty acids and to ketonize the hydroxyl group, and it produces various intermediary metabolites including 10-hydroxy-*cis*−12-octadecenoic acid (HYA) through these conversions [6]. HYA is a stronger agonist of GPR40 and GPR120 than linoleic acid [3, 7], and it attenuates inflammatory responses in CACO-2 and BV-2 cells [7, 8]. Converting dietary linoleic acid to HYA by gut microbiota suppresses the production of inflammatory mediators [3]. HYA might therefore be superior to linoleic acid at the point of the agonistic activity of GPR40 and GPR120 and inflammatory responses.

Previous reports have indicated that the oral administration of HYA 2 h before OGTT in mice ameliorated postprandial hyperglycemia as well as linoleic acid, depending on the promotion of insulin secretion via GPR40 pathway [3]. Although the oral administration of HYA immediately before OGTT was expected to reveal the amelioration of postprandial hyperglycemia by the same mechanism as linoleic acid, as reported in our previous study, the result was unclear [4]. For clarification, we now investigate the effect of orally-administration of HYA immediately before OGTT on postprandial glucose level in rats.

## Materials and methods

### Chemicals

[^14^C]-Labelled methyl-α-D-glucopyranoside ([^14^C] α-MDG; 50 µCi; 1.85 MBq; 250 mCi/mmol) was purchased from Perkin Elmer (Waltham, MA, USA). Phlorizin was from FUJIFILM Wako Pure Chemical Corp. (Osaka, Japan). HYA were synthetized from linoleic acid by Noster Inc. (Kyoto, Japan), which has been detailed in previous studies [3, 7, 8]. Sterilized olive oil was obtained from Yoshida Pharmaceutical Co., Ltd (Tokyo, Japan), while human insulin (Humulin^®^ R) was purchased from Eli Lilly (Indianapolis, IN, USA).

## Animals

Five-week-old male Sprague-Dawley rats were from CLEA Japan, Inc. (Tokyo, Japan). Rats were housed in plastic cages (24.7 × 40.9 × 19.7 cm) with free access to laboratory animal chow (Oriental Yeast Co., Ltd., Tokyo, Japan) and tap water under a 12 h light/dark cycle (lights on at 8:00 AM) at 25 ± 1 ºC and 50–60% humidity. All animals were used after a one-week acclimation period. The protocol of animal experiments was approved by the Wakayama Medical University Animal Experiment Committee on July 1, 2019 (No. 883).

## Cell culture

CACO-2 cells were from Deutsche Sammlung für Mikroorganismen und Zellkulturen (Braunschweig, Germany). They were maintained in Dulbecco’s modified Eagle’s medium supplemented with 10% fetal bovine serum, 1% non-essential amino acids, and 100 IU/mL penicillin-100 µg/mL streptomycin. Cells were grown under 5% CO_2_−95% air at 37 °C.

## Glucose tolerance testing and measurement of glucose and insulin

OGTT was performed as in the previous reports [4, 9]. Rats were fasted overnight for performance of OGTT (2 g/kg body weight), and they were administered HYA solution (2.5 g/5 mL) or olive oil (each 5 mL/kg bodyweight) immediately before OGTT. Blood was taken from the tail vein prior to the OGTT and then 30, 60 and 120 min after the OGTT. Blood glucose levels were directly measured from blood samples with Freestyle Libre (Abbott, Alameda, CA). Concentration of insulin in plasma was measured using rat insulin measurement kits (Morinaga Institute of Biological Science, Kanagawa, Japan).

## [^14^C] α-MDG uptake study

[^14^C]α-MDG uptake study was performed as previously reported [4], the procedures are summarized below. CACO-2 cells were seeded at a cell density of 2.0 × 10^5^ cells/well on 24-well plastic plates. The culture medium was refreshed every other day, and the cell monolayers were used for the uptake experiments 14 days after plating. The culture medium was removed and then all wells were washed and pre-incubated with glucose-free Hank’s balanced salt solution (HBSS) buffer, and then 0.5 mL of HBSS buffer containing 0.4 µM (0.1 µCi/mL). [^14^C] α-MDG in the absence or presence of various compounds was added and incubated for 30 min at 37 °C. Each cell monolayer was rapidly washed twice with 1.0 mL ice-cold HBSS buffer at the end of the incubation period. To quantify the radioactivity of [^14^C] α-MDG, the cells were solubilized in SDS buffer, then to estimate the radioactivity, the remaining sample was mixed with 3 ml of scintillation cocktail. Uptake values were corrected for protein content, and the protein concentration was determined using a Pierce^®^ BCA Protein Assay Kit (Thermo Fisher Scientific, Waltham, MA, USA).

### Measuring glucose in the stomach after OGTT

OGTT was performed according to the above protocol. Rats were anesthetized with isoflurane 30 min after the OGTT, and the stomachs were collected after ligating at the cardia and the pylorus. Gastric content was then collected in cold phosphate-buffered saline. The gastric content suspensions were centrifuged at 400 g for 10 min, and after measuring the volume of the supernatant, the concentration of glucose within it was measured using Lab Assay Glucose (FUJIFILM Wako Pure Chemical Corporation). The remaining amounts of glucose in the gastric tract were calculated from the volume and concentration of glucose in the supernatant, and the residual ratio of glucose in the gastric tract was calculated in relation to the amount of glucose administrated in OGTT. Blood was collected from tail veins before OGTT and before anesthesia so that we could measure the concentration of GLP-1 and cholecystokinin (CCK) in plasma. These concentrations were measured using rat GLP-1 ELISA kit (FUJIFILM Wako Pure Chemical Corporation) and rat CCK Enzyme Immunoassay Kit (RayBiotech, Norcross, GA, USA), respectively. Blood samples from the portal vein were also collected under anesthesia, and the concentration of glucose in the serum was measured with Lab Assay Glucose (FUJIFILM Wako Pure Chemical Corporation).

## Glucose tolerance testing and measurement of glucose in type 1 diabetes model rats

Type 1 diabetes model rats were made using previously-reported methods [4]. To prepare the type 1 diabetes model rats, six-week-old male Sprague-Dawley rats were intraperitoneally injected with 60 mg/kg streptozocin on the first day and 100 mg/kg streptozocin on the second day. The following week, the concentrations of plasma glucose and insulin were measured before and 30 min after oral glucose load (2 g/kg body weight). We excluded the rats according to two criteria in this experiment: blood glucose level < 500 mg/dL 30 min after glucose load, and the concentration of plasma insulin increased 1.5-fold 30 min after glucose load compared with that before glucose load. OGTT and the measurement of glucose level were also performed according to the above protocol in type 1 diabetes rats. The type 1 diabetes model rats given bolus insulin were also subjected to OGTT following the subcutaneous injection of Humulin^®^ R (0.1 U/kg).

### Statistical analysis

All data are presented as the means ± SD. Statistical analyses were by JMP 14 (SAS Institute, Cary, NC). Repeated measures analysis of variance (ANOVA) was used in analysis of concentration of blood glucose and plasma insulin changes after OGTT. Student’s t test was used to compare two groups. In comparison of three or more groups, Dunnett’s test after a one-way ANOVA was performed in comparison with the control group. Wilcoxon’s test were performed to compare the time to elevate the postprandial blood glucose level up to the peak in the control and HYA groups. Significance levels were set at 0.05 in this study.

## Results

### Effects of HYA administration on the blood glucose level in rats

**Fig. 1:**
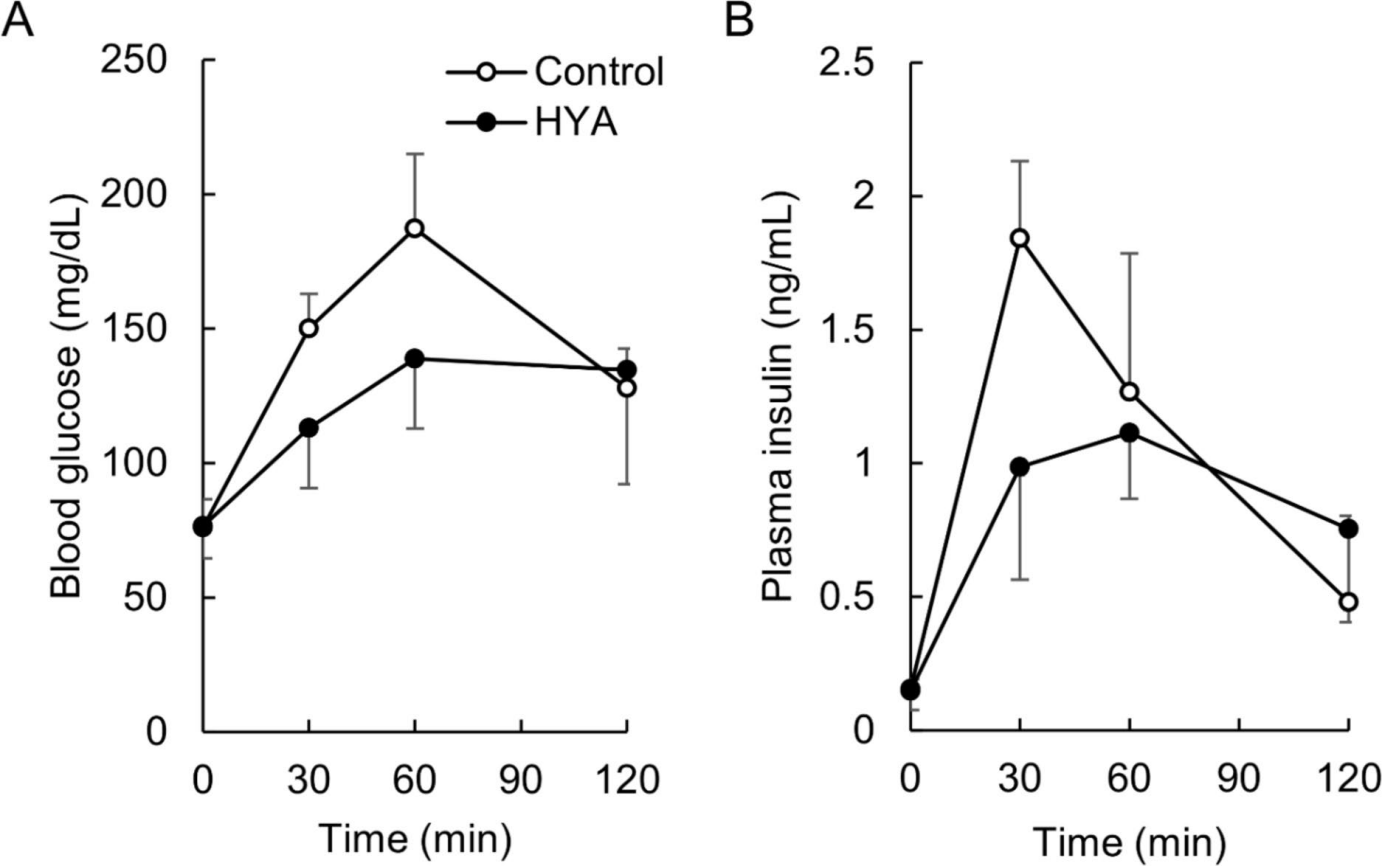
HYA ameliorated postprandial hyperglycemia without promoting the secretion of insulin. The blood glucose level (**A**) and plasma insulin level (**B**) after OGTT in rats were analyzed immediately after oral HYA administration (2.5 g/kg body weight). Values indicate mean ± SD. *n* = 4 to 6

We examined whether orally-administered HYA immediately before the OGTT slowed the elevation of postprandial blood glucose level in the normal rats (Fig. 1). Repeated measures ANOVA indicated that the effect of the administration of HYA on the blood glucose level was significant [F (1, 10) = 10.5, *p* < 0.05]. The effect of time on the blood glucose level [F (3, 30) = 26.76, *p* < 0.05] and their interaction was significant [F (3, 30) = 3.84, *p* < 0.05]. The glucose levels were lower in the HYA group 30 and 60 min after OGTT than in the control group (Fig. 1A). Repeated measures ANOVA also indicated that the effect of HYA was dose-dependently observed [F (4, 21) = 8.62, *p* < 0.05] (Supplemental Fig. 1A). Orally-administered HYA alone did not decrease the blood glucose level in normal rats (Supplemental Fig. 1B). Repeated measures ANOVA indicated that the effect of insulin concentration in the plasma on the administration of HYA was not significant [F (1, 7) = 3.52, *p* = 0.10]. However, the effect of time was significant [F (3, 21) = 25.46, *p* < 0.05], as was their interaction [F (3, 21) = 4.74, *p* < 0.05]. The concentration of insulin in plasma was lower 30 min after OGTT in the HYA group than in the control group (Fig. 1B).

### HYA inhibited the uptake of glucose in SGLT1 and slowed gastric motility

**Fig. 2:**
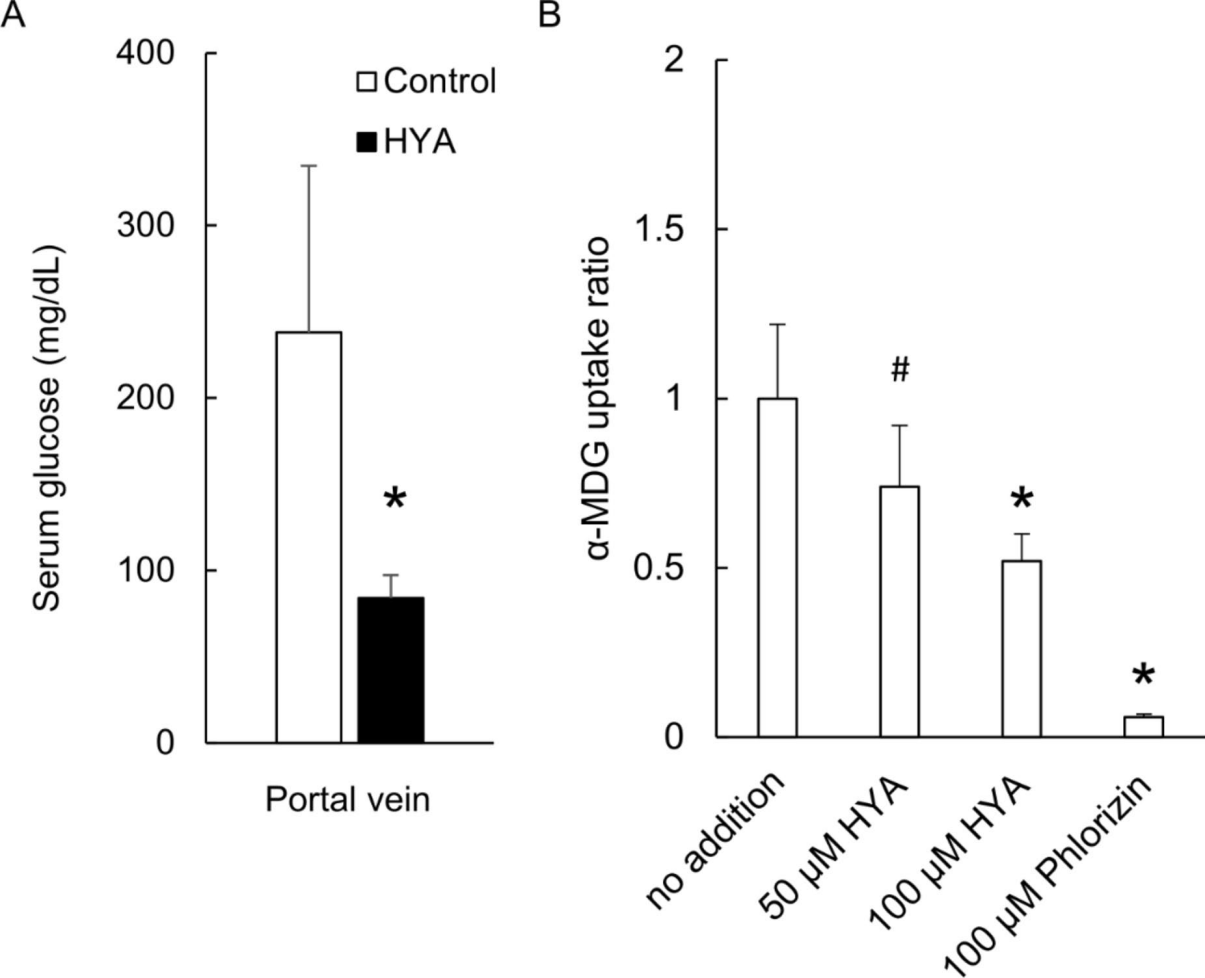
The partial inhibition of HYA on the absorption of glucose in the mucosa of the small intestines via SGLT1. The serum glucose levels in the portal vein 30 min after OGTT were measured immediately after oral HYA administration (**A**). The uptake ratio of α-MDG for 30 min in CACO-2 cells treated by HYA or phlorizin were analyzed and compared with no treatment (**B**). Values indicates mean ± SD. *n* = 4 to 6. Asterisks indicate significant difference compared with control group (*p* < 0.05). # indicates that the *p* value was 0.06

To clarify the mechanism of ameliorating postprandial hyperglycemia in HYA administration, we measured the portal blood glucose level 30 min after OGTT. The portal blood glucose levels were lower in the HYA group than in the control group (*p* < 0.05, Fig. 2A), which indicated that HYA might inhibit the absorption of glucose in the intestinal mucosa.

The inhibition of glucose absorption might be associated with the inhibition of the glucose uptake via sodium-dependent glucose transporter (SGLT) 1 in the rat intestinal mucosa. We examined whether HYA inhibited the uptake of α-MDG, which is transported by SGLT1, in CACO-2 cells treated by HYA. One-way ANOVA indicated that there was significantly different uptake of α-MDG between the four groups (no addition, 50 µM HYA, 100 µM HYA, 100 µM phlorizin) (F (3,12) = 28.9, *p* < 0.05). Dunnett’s test indicated that phlorizin, an SGLT1 inhibitor, inhibited the uptake of α-MDG (*p* < 0.05) compared with when it was not added. The uptake of α-MDG was significantly inhibited by 100 µM HYA (*p* < 0.05) and tended to be inhibited by 50 µM HYA (*p* = 0.06). HYA therefore dose-dependently inhibited the uptake of α-MDG (Fig. 2B).

**Fig. 3:**
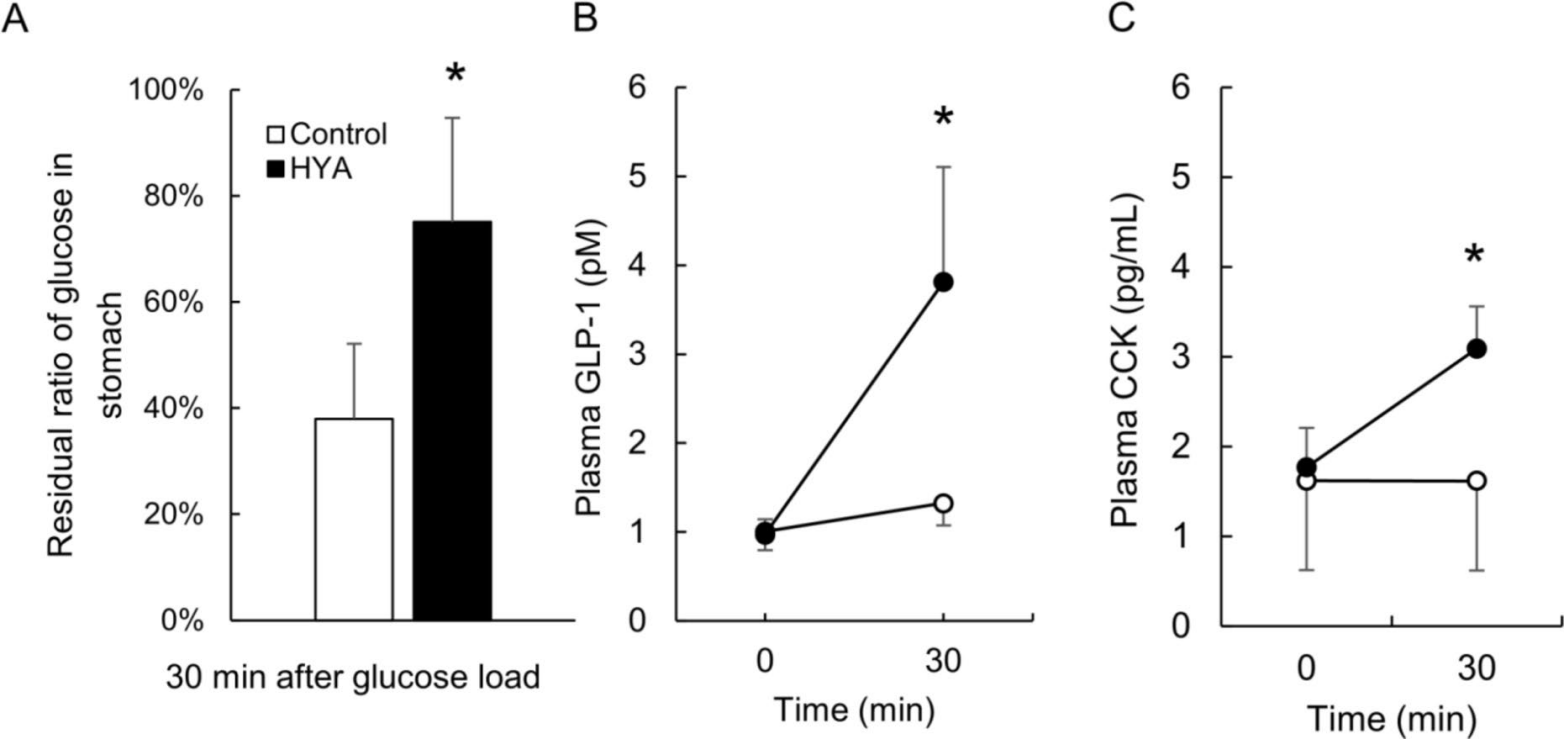
HYA slowed the gastric motility by promoting the secretion of CCK and GLP-1. The residual ratio of glucose in the stomach 30 min after OGTT in rats was compared with the amount of oral glucose administered (**A**). The plasma GLP-1 (**B**) and CCK (**C**) levels before or 30 min after OGTT were measured. Values indicates mean ± SD. *n* = 4 to 6. Asterisks indicate significant difference from the control group at same time point (*p* < 0.05)

To examine whether the amelioration of postprandial hyperglycemia in HYA administration might be caused by slowing gastric motility, we measured the amount of glucose in the gastric tract 30 min after the OGTT (Fig. 3A). The residual ratio of glucose in the stomach was significantly higher in the HYA group than in the control group at 30 min (*p* < 0.05), which indicates that HYA slowed the transportation of glucose from the stomach to the duodenum. To clarify this mechanism, we measured the plasma concentrations of GLP-1 and CCK, which are intestinal hormones that suppress gastric motility. The plasma concentrations of GLP-1 and CCK were significantly increased in the HYA group 30 min after OGTT compared with in the control group (*p* < 0.05) (Fig. 3B and C).

### Beneficial effects of HYA on postprandial glucose level in type 1 diabetes model rats

**Fig. 4:**
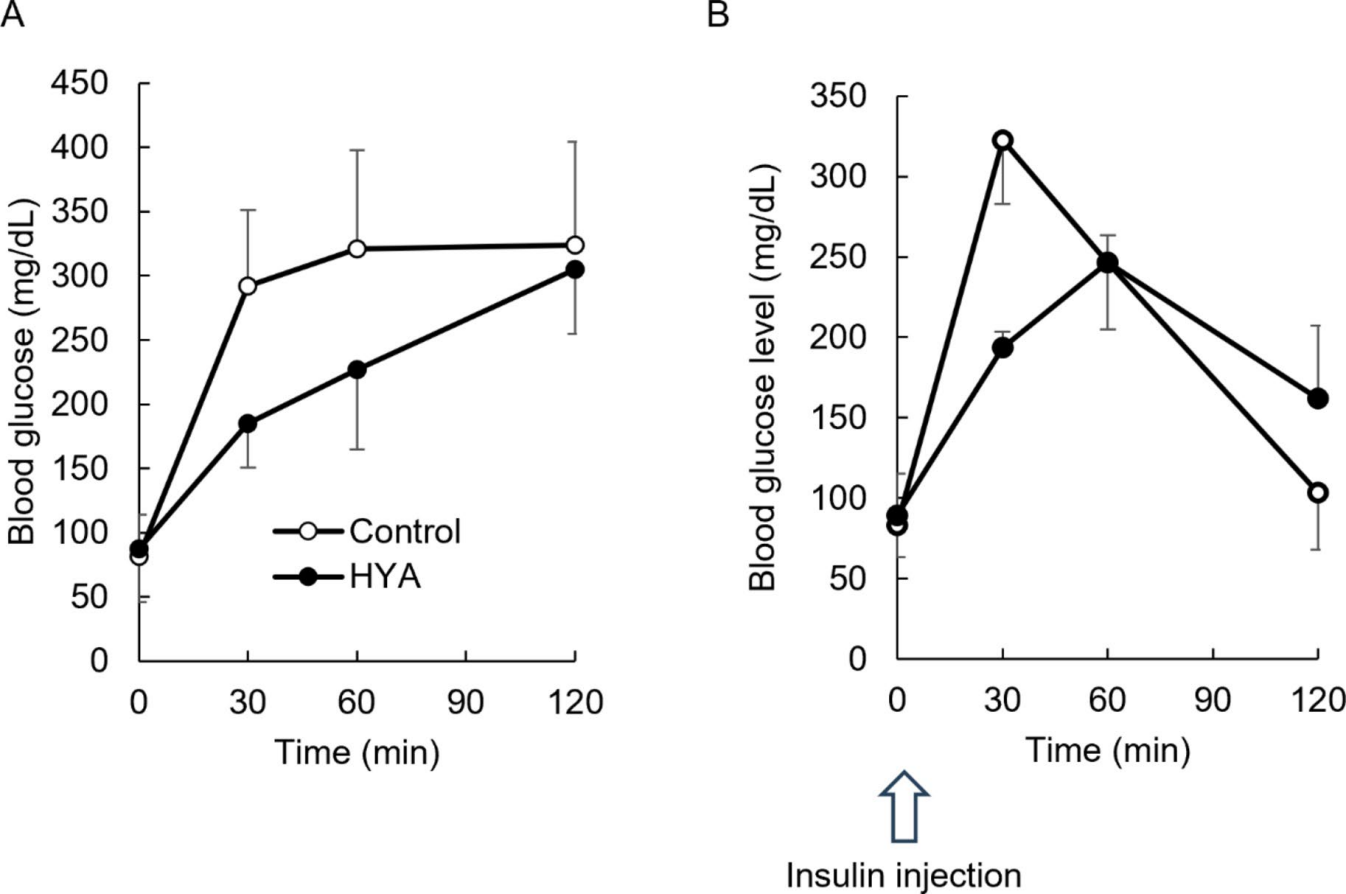
Oral administration of HYA immediately before OGTT also ameliorates the postprandial hyperglycemia in type 1 diabetes rats. The blood glucose level after OGTT in type 1 diabetes rats (**A**) and type 1 diabetes rats given bolus insulin subcutaneously (**B**) were analyzed immediately after oral HYA administration. Values indicates mean ± SD. *n* = 4 to 6

This beneficial effect of HYA might be associated with the inhibition of glucose uptake and suppression of gastric motility rather than the promotion of insulin secretion (Figs. 1B, 2 and 3). To clarify whether the effect occurred without promoting insulin secretion, we examined whether the beneficial effect of HYA also occurred in type 1 diabetes model rats (Fig. 4). Repeated measured ANOVA indicated that the effect of HYA on the blood glucose level was significant [F (1, 10) = 9.44, *p* < 0.05]. The effects of time [F (3, 30) = 56.89, *p* < 0.05] and the interaction [F (3, 30) = 10.12, *p* < 0.05] were also significant. The elevation of postprandial glucose levels was slower in the HYA group than in the control group.

Furthermore, we examined whether HYA ameliorated postprandial hyperglycemia in the type 1 diabetes model rats administered bolus insulin immediately before OGTT. Repeated measured ANOVA indicated that the effect of HYA on the blood glucose level was not significant [F (1, 6) = 0.75, *p* = 0.42]. The effect of time was significant [F (3, 18) = 130.45, *p* < 0.05] and their interaction was also significant [F (3, 18) = 28.91, *p* < 0.05]. The postprandial glucose levels were lower in the HYA group than the control group 30 min after OGTT (Fig. 4B).The peaks of postprandial glucose levels were statistically lower in the HYA group than in the control group (control: 322.5 ± 39.5 mg/dL, HYA: 246.5 ± 16.8 mg/dL, *p* < 0.05), and the time to elevate postprandial glucose levels up to the peak was statistically prolonged in the HYA group compared with in the control group (*p* < 0.05) (Fig. 4B).

## Discussion

Oral administration of HYA immediately before OGTT slowed the elevation of postprandial glucose levels in normal rats (Fig. 1A, Supplemental Fig. 1) as well as oral administration of linoleic acid [4]. This beneficial effect was associated without promotion of insulin secretion (Figs. 1B and 4A) but with inhibition of the absorption of glucose in the small intestine (Fig. 2A). HYA inhibited SGLT1-mediated transport in CACO-2 cells (Fig. 2B). It also slowed the gastric motility following the promotion of secretion of GLP-1 and CCK in normal rats (Fig. 3). Furthermore, HYA ameliorated postprandial hyperglycemia in type 1 diabetes model rats injected with bolus insulin immediately before OGTT (Fig. 4B). These results suggest that the beneficial effect of HYA might be associated with the inhibition of glucose uptake via SGLT1 in the mucosa of small intestine, and the slowing of transportation of glucose from the stomach to the small intestine via promotion of GLP-1 and CCK secretion.

SGLT1 is expressed on the brush-border membrane of epithelial cells from the small intestine to take up the glucose in the intestinal tract [10, 11]. HYA inhibited glucose uptake via SGLT1 in CACO-2 cells, although the inhibition was weaker than phlorizin, an SGLT1 inhibitor (Fig. 2B). LX2761 and LC-4211, SGLT1 inhibitors, inhibited the elevation of blood glucose level after OGTT through inhibiting the absorption of glucose in the small intestine mucosa [12, 13]. In patients with type 1 diabetes, sotagliflozin, a dual SGLT1 and SGLT2 inhibitor, improved glycemic control [14]. The amelioration of postprandial hyperglycemia in HYA might therefore be partially associated with SGLT1 inhibition.

In the current study, approximately double the amount of glucose remained 30 min after glucose load in the stomachs of the HYA group compared with in the control group (Fig. 3A). GLP-1 and CCK, which slow the gastric motility, are secreted in the small intestine via GPR120 [15, 16]. HYA acted as an agonist of GPR120 and promoted the secretion of GLP-1 [3]. The plasma levels of GLP-1 and CCK 30 min after the OGTT were increased by administration of HYA (Fig. 3B and C). Gastric motility slowed by HYA might therefore be associated with the promotion of secretion of GLP-1 and CCK via GPR120 in normal rats. The peak of postprandial hyperglycemia was lower in the patients with diabetes whose gastric emptying was lower [17], and GLP-1 and CCK were secreted after a meal in the patients with type 1 diabetes [18, 19]. This beneficial effect of HYA on postprandial hyperglycemia might therefore be associated with slowing gastric motility through promotion of the secretion of GLP-1 and CCK.

Several oral diabetes agents are known to promote the secretion of insulin in the pancreas, but they can cause hypoglycemia [20, 21]. Novel diabetes drugs have recently been developed, and the mechanisms of these drugs are enhancing insulin secretion responding to postprandial hyperglycemia through GLP-1 signaling [22, 23] and inhibition of glucose absorption via SGLT2 [24, 25]. These types of drugs have lower risks of hypoglycemia, because the secretion of insulin is not directly induced. Oral-administration of HYA without OGTT did not decrease the blood glucose level in normal rats (Supplemental Fig. 1B). This study demonstrated that HYA might be a diabetes agent without accompanying a risk of hypoglycemia.

Orally-administered HYA immediately before OGTT slowed the elevation of postprandial glucose level in type 1 diabetes model rats (Fig. 4A). In this study, we used type 1 diabetes model rats injected with bolus insulin. This is similar to the situation before a meal for patients with type 1 diabetes. The peak of postprandial glucose level was lower in the HYA group than in the control group in this type 1 diabetes model (Fig. 4B). Inhibition of hyperglycemia is important because it is associated with cardiovascular disorders, peripheral neuropathy, and diabetic retinopathy [26–29]. The administration of rapid-acting insulin analogs 15–20 min before meals reportedly ameliorated postprandial hyperglycemia compared with administration immediately before meals in patients with type 1 diabetes [30, 31]. Slowing of gastric motility induced by HYA might be a useful proxy for the administration of bolus insulin 15–20 min before a meal. This might have possible application as a novel treatment for patients with type 1 diabetes whose postprandial hyperglycemia would be difficult to control.

## Conclusion

Orally-administered HYA before OGTT slowed the elevation of blood glucose level after OGTT due to inhibiting glucose absorption via SGLT1 in small intestine mucosa and slowing the transportation of glucose from the stomach to the duodenum via promotion of GLP-1 and CCK secretion in normal rats. HYA also ameliorated postprandial hyperglycemia in type 1 diabetes model rats injected with bolus insulin before OGTT.

## Supplementary Information

Below is the link to the electronic supplementary material.

Supplementary file1 (TIFF 896 KB) Effect of HYA on blood glucose levels after glucose load in dose-dependent manner and in rats not loading glucose. The postprandial hyperglycemia was ameliorated by HYA in a dose-dependent manner (A). The transition of blood glucose level without glucose load after oral HYA administration was not decreased (B). Values indicates mean ± SD. n = 4 to 6.

## Declarations

### Research involving human and animal participant

All procedures performed in studies involving human participants were in accordance with the ethical standards of the institutional and/or national research committee and with the 1964 Helsinki Declaration and its later amendments or comparable ethical standards. The protocol of animal experiments was approved by the Wakayama Medical University Animal Experiment Committee on July 1, 2019 (No. 883).
