# Supplementary figures and images for: HYA ameliorated postprandial hyperglycemia in type 1 diabetes model rats with bolus insulin treatment
Source: Acta Diabetol. 2025 Feb 3;62(8):1337–45. doi: 10.1007/s00592-025-02459-6 (PMC12364981; doi:10.1007/s00592-025-02459-6)

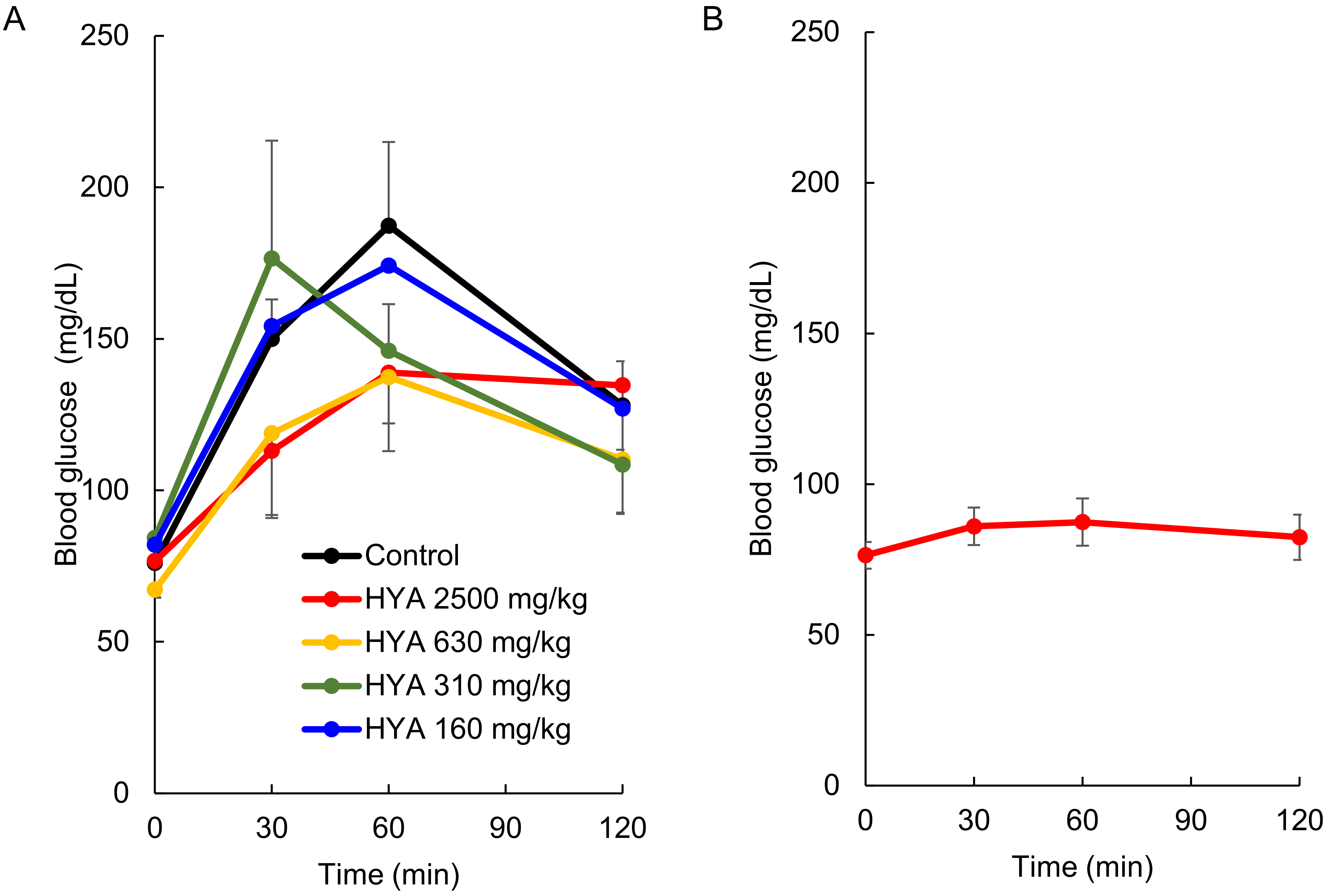

Supplement: Supplementary file 1 — Supplementary file1 (TIFF 896 KB) Effect of HYA on blood glucose levels after glucose load in dose-dependent manner and in rats not loading glucose. The postprandial hyperglycemia was ameliorated by HYA in a dose-dependent manner (A). The transition of blood glucose level without glucose load after oral HYA administration was not decreased (B). Values indicates mean ± SD. n = 4 to 6. [file 592_2025_2459_MOESM1_ESM.tif]
